# Applicability of LDL-cholesterol calculation formulas in hypertriglyceridemia: Insights from the Vojvodina

**DOI:** 10.5937/jomb0-61948

**Published:** 2026-06-16

**Authors:** Dragana Žuvić, Stanislava Nikolić, Romana Mijović, Branislava Ilinčić, Aneta Ranđelović-Živković, Dušan Sedlarević, Velibor Čabarkapa

**Affiliations:** 1 University of Novi Sad, Center for Laboratory Diagnostics, University Clinical Center of Vojvodina, Faculty of Medicine, Novi Sad

**Keywords:** lipids, cholesterol, LDL, hypertriglyceridemia, Sampson formula, lipidi, holesterol, LDL, hipertrigliceridemija, Sampsonova formula

## Abstract

**Background:**

Low-density lipoproteins cholesterol (LDL-C) is a key parameter for assessing the risk of atherosclerotic cardiovascular disease. Direct measurement of LDL-C is not always possible due to practical or financial reasons, making the use of calculation formulas essential. The aim of this study was to evaluate the applicability of four different formulas proposed by Friedewald, Sampson, Anandaraja, and Martin-Hopkins for calculating LDL-C compared to direct method in patients with serum triglyceride levels from 4.5 to 9.0 mmol/L in the population of Vojvodina.

**Methods:**

The retrospective study included 272 subjects whose lipid status parameters were measured between June 2022 and June 2023. LDL-C was determined by the direct method (d-LDL-C) on the Alinity c analyser (Abbott Laboratories, Illinois, USA). Calculated LDL-C values were obtained using the four selected formulas.

## Abstract (sr)

**Uvod:**

LDL-holesterol (LDL-C) predstavlja kljucni parametar u proceni rizika od nastanka aterosklerotske kardiovaskularne bolesti. Određivanje LDL-C direktnom metodom nije uvek moguće iz praktičnih ili finansijskih razloga, zbog čega izračunavanje pomoću formula ima značajnu ulogu u njegovoj proceni. Cilj ovog istraživanja bio je da se ispita primenljivost četiri različite formule, koje su predložili Friedewald, Sampson, Anandaraja i Martin-Hopkins, za izračunavanje LDL-C u poređenju sa direktnom metodom kod pacijenata sa vrednostima serumskih triglicerida između 4,5 i 9,0 mmol/L u populaciji Vojvodine.

**Metode:**

Istraživanje je sprovedeno kao retrospektivna studija koja je ukljudla 272 ispitanika kod kojih su parametri lipidnog statusa određivani u periodu od juna 2022. do juna 2023. godine. LDL-C je određivan direktnom metodom (d-LDL-C) na analizatoru Alinity c (Abbott Laboratories, Illinois, SAD), dok su vrednosti LDL-C dodatno izračunavane primenom četiri navedene formule.

**Rezultati:**

Prosečna starost ispitanika iznosila je 52 godine, dok je medijana koncentracije triglicerida bila 5,48 (4,94-6,58) mmol/L. Utvrđena je statistički značajna pozitivna korelacija između d-LDL-C i svih izračunatih vrednosti LDL-C (p&lt; 0,001). Linearna regresiona analiza pokazala je najbolje slaganje između d-LDL-C i Sampsonove formule (slope=0,97; 95% CI = 0,91-1,04). Najmanja prosečna razlika (MD) uočena je između d-LDL-C i LDL-C izračunatog prema formuli Sampsona i saradnika (MD=-0,032). Poređenjem d-LDL-C sa izračunatim vrednostima LDL-C, jedino formula Sampsona i saradnika nije pokazala statistički značajnu razliku (P=0,240).

**Zaključak:**

U ispitivanoj populaciji pacijenata sa hipertrigliceridemijom, formula Sampsona i saradnika pokazala je najbolje performanse u poređenju sa ostalim ispitivanim formulama.

## Introduction

Lipoproteins are spherical molecules in the blood that transport cholesterol (CHOL) and triglycerides (TG) to cells and tissues [1]. Their importance to the human body is reflected in their roles in energy metabolism, lipid deposition, and the synthesis of steroid hormones and bile acids [2]. Low-density lipoproteins transport most of the cholesterol from the liver to peripheral tissues [3]. Determination of certain lipoproteins in the blood has been used as a marker for cardiovascular risk assessment for a long time. Numerous studies have shown the association between low-density lipoproteins-cholesterol (LDL-C) serum levels and the risk of developing atherosclerotic cardiovascular disease (ACVD) [3]. LDL-C is the main parameter assessed for ACVD occurrence, and its routine determination is recommended as a major target for monitoring and treating patients with hyperlipidemia [1][2].

The reference method for measuring LDL-C is beta-quantification, which involves ultracentrifugation to separate lipoprotein particles [4]. However, this method is impractical for routine clinical use due to its time-consuming nature, high cost, requirement for large sample volumes, and the need for specialized equipment such as an ultracentrifuge, limiting its application to specialized laboratories [5].

In clinical practice, LDL-C serum concentration is typically determined using direct chemical assays or indirectly calculated using various formulas [5]. Direct measurement (d-LDL-C) is performed using homogeneous, fully automated assays known for their excellent accuracy; however, they are often not optimized for low LDL-C levels [6]. In developing countries with limited resources, many laboratories use a simpler, cheaper method to calculate LDL-C indirectly using various formulas [3].

The most commonly used formula is the Friedewald equation (F-LDL-C), which allows us to estimate the cholesterol concentration in the atherogenic LDL fraction in a relatively simple manner. This calculation is based on directly measured concentrations of CHOL, TG, and high-density lipoproteins-cholesterol (HDL-C) [7]. However, various studies have shown that the F-LDL-C cannot be applied when the serum TG concentration is above 4.5 mmol/L, when the samples were not taken in a fasting state, or in type III hyperlipidaemia [5].

Due to the factors mentioned above, the accuracy of direct measurement, as well as calculations, plays a crucial role in determining precise LDL-C values. Many researchers have tried to modify the Friedewald equation to overcome its limitations. Overestimation and underestimation of LDL-C values can be a significant issue for patients. Overestimation may lead to unnecessary drug prescriptions, in addition to being expensive, can also cause numerous side effects, while underestimating LDL-C levels may delay necessary treatment, thereby increasing cardiovascular risk in affected patients [3].

There are several published equations for calculation of LDL-C, but most of them are designed for use in patients with triglycerides below 4.5 mmol/L [7][8][9][10]. Each formula provides different results, and it remains unclear which should be preferred, especially for cardiovascular risk assessment.

The Sampson and colleagues-National Institutes of Health Equation 2 (S-LDL-C), proposed in 2020, was developed using beta-quantification and multiple least squares regression to calculate very low-density lipoproteins (VLDL) in a population with high TG [8]. Specifically, the formula by Sampson et al. aimed to provide the most accurate estimation of LDL-C in patients with very low LDL-C levels and those with hypertriglyceridemia [11]. It demonstrated good performance in populations with TG levels up to 9.0 mmol/L [8]. The advantages of formula of Sampson and colleagues, include its simplicity of calculation and application, as well as significant financial savings for laboratories, as it does not require application of an assay for direct measurement of LDL-C [5].

The formula proposed by Anandaraja et al. (A-LDL-C) in the Indian population has not been evaluated in patients with hypertriglyceridemia, so additional studies are needed to assess its use in this population [9].

Martin-Hopkins formula (MH-LDL-C) for calculating LDL-C was developed using an adjustable factor for the TG/VLDL-cholesterol ratio, based on TG and NON-HDL concentrations [10]. Its primary goal was to improve alignment with individual patient classification into appropriate cardiovascular risk categories. This formula has demonstrated greater precision than F-LDL-C in classifying LDL-C concentration below 1.8 mmol/L in patient with elevated TG levels up to 4.5 mmol/L. The authors noted that this formula is not suitable for use in cases of severe hypertrygliceridemia and type III dyslipidemia [10]. One limitation of the Martin-Hopkins formula is the complexity of implementing the 180-cell stratification in a laboratory information system [5].

With the increasing prevalence of overnutrition and obesity accompanied by hypertriglyceridemia, it is essential to have an accurate formula to determine LDL-C levels precisely in this patient group, without requiring use of direct assays. This would ultimately contribute to more effective therapy in preventing ACVD [4].

Our study included participants with TG levels above 4.5 mmol/L, as the Friedewald formula is insufficient beyond that point, whereas the Sampson formula showed it can be adequate up to 9.0 mmol/L.

The aim of this study was to examine the applicability of four different formulas, according to the authors Friedewald, Sampson, Anandaraja, and Martin-Hopkins for calculating LDL-C compared to direct method in patients with serum triglyceride levels from 4.5 to 9.0 mmol/L in the population of Vojvodina, the northern province of Serbia.

## Materials and methods

This study was retrospective. Data on lipid status parameters were collected from the Laboratory information system database of the Center of Laboratory Diagnostic, University Clinical Center of Vojvodina for a period from June 2022 to June 2023.

We analyse data parameters of lipid status from 272 subjects older than 18 years, including 179 men and 93 women, with serum TG levels between 4.5 mmol/L and 9.0 mmol/L. Venous blood was drawn from the cubital vein of fasting patients into collection tubes with serum separator. After centrifugation, the serum was immediately used to determine lipid profile parameters.

Lipid status assessment included measurements of ChOl, TG, HDL-C, and d-LDL-C serum levels on an Alinity c (Abbott Laboratories, Illinois, USA) automated analyser. d-LDL-C was measured with Alinity c Direct LDL homogeneous assay. The method is in two reagent formats, based on a liquid- selective detergent. From these parameters, we calculated NON-HDL cholesterol (NON-HDL) using the formula: NON-HDL=(CHOL)-(HDL-C).

For easier application of the formulas, CHOL, HDL-C, d-LDL-C and NON-HDL values were converted from mmol/L to mg/dl by multiplying by 38.67, and TG values were converted from mmol/L to mg/dl by multiplying by 88.57.

In addition to the direct determination of LDL-C, we also calculated LDL-C using the following formulas:

Friedewald: F-LDL-C=(CHOL)-(HDL-C)-TG/5 [7],

Sampson: S-LDL-C=(CHOL/0,948)-(HDL-C/0,971)-(TG/8,56+TG*NON-HDL/2140-TG2/ 16100)-9,44 [8],

Anandaraja: A-LDL-C=(0,9*CHOL)-(0,9*TG/5)-28 [9],

Martin-Hopkins: MH-LDL-C=(CHOL-(HDL-C)-TG)/adjustable factor derived using the Johns Hopkins University calculator [10].

### Statistical analysis

Statistical analysis was performed using JASP 0.19.1 statistical software (JASP [Computer software]). The Shapiro-Wilk and Kolmogorov-Smirnov tests were used to test the normality of the distribution. Variables with a normal distribution are presented as mean ± standard deviation (Mean(SD)), while variables with a non-normal distribution are presented as median and interquartile range (Me(IQR: Q1-Q3)). Since our variables, d-LDL-C and calculated LDL-C, were not normally distributed, a Spearman correlation analysis was performed to assess their association. To enable linear regression analysis, a logarithmic transformation was applied to normalize the data. After log transformation, linear regression analysis was used to evaluate the relationship and agreement between the direct and calculated LDL-C values. The Bland-Altman plots were also performed, and the mean difference (MD) between d-LDL-C and calculated LDL-C was determined. The Friedman test was used to compare d-LDL-C concentrations with values calculated using different formulas within the same participants. When a significant overall difference was observed, Conover's post hoc test was applied to determine pairwise differences between measurement methods. The chosen level of significance is: statistically significant P-value<0.05.

According to the recommended target values based on cardiovascular risk assessment, we divided LDL-C into five groups (<1.4 mmol/L, 1.4-1.8 mmol/L, 1.8-2.6 mmol/L, 2.6-3.0 mmol/L, >3.0 mmol/L) in accordance with the latest European guidelines [2]. Contingency tables were used to evaluate the misclassification rate of LDL-C across these categories.

The study was approved by the Ethics Committee of the University Clinical Center of Vojvodina (15 August 2024, number 00-271).

## Results

The study included 272 subjects, comprising 179 men (65.8%), and 93 women (34.2%), with measured serum triglyceride levels ranging from 4.5 to 9.0 mmol/L. The average age of the subjects was 52 years. The youngest participant had 23 years and the oldest was 83 years old. Table 1 presents the lipid status parameter values in the examined population.

**Table 1:** Lipid status parameters in the examined population. Me=median, IQR = interquartile range: Q1-Q3, SD=standard deviation, CHOL=total cholesterol, TG=triglyceride,HDL-C = high-density lipoprotein cholesterol, NON-HDL=NON-HDL cholesterol, d-LDL-C= low-density lipoprotein-cholesterol determined by direct method

	n=272 <br>Me (IQR) <br>Mean (SD)	Men n = 179 <br>Me (IQR) <br>Mean (SD)	Women n = 93 <br>Me (IQR) <br>Mean (SD)
CHOL (mmol/L)	6.40 (5.40-7.68)	6.28 (5.26-7.55)	6.79±1.70
TG (mmol/L)	5.48 (4.94-6.58)	5.50 (4.97-4.83)	5.46 (4.83-6.41)
HDL-C (mmol/L)	0.85 (0.25)	0.82 (0.25)	0.90 (0.25)
NON-HDL (mmol/L)	5.48 (4.50-6.71)	5.39 (4.48-6.69)	5.88 (1.63)
d-LDL-C (mmol/L)	3.26 (2.52-4.14)	3.12 (2.39-6.84)	3.50 (1.26)

**Figure 1:**
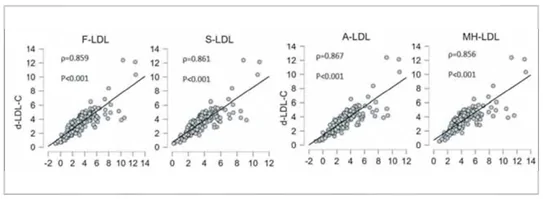
Spearman correlation analysis between d-LDL-C and calculated LDL-C.

Figure 1 presents the Spearman correlation analysis between d-LDL-C and LDL-C, calculated using different formulas. A statistically significant positive correlation was found between d-LDL-C and all calculated LDL-C values (P<0.001). The highest correlation coefficient with d-LDL-C was demonstrated the A-LDL-C (r=0.867), whereas the F-LDL-C, S-LDL-C, and MH-LDL-C showed slightly lower correlation coefficients (r=0.859, r=0.861, r=0.856, respectively).

**Figure 2:**
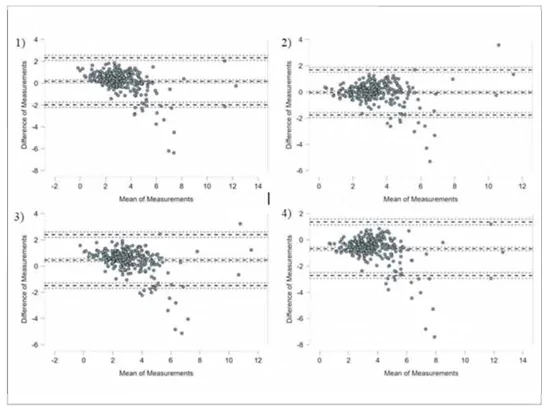
Bland-Altman Graphics between d-LDL-C and 1) F-LDL-C, 2) S-LDL-C, 3) A-LDL-C, 4) MH-LDL-C.

Mean difference ± 1.96SD was calculated for each formula compared to d-LDL-C (Table 2). S-LDL-C showed the lowest MD. Bland-Altman graphics were performed (Figure 2).

**Table 2:** Mean difference between d-LDL-C and calculated values. MD=mean difference, 95%CI = 95% confidence interval, SD=standard deviation, d-LDL-C = low-density lipoprotein-cholesterol determined by direct method, F-LDL-C = LDL-C calculated using Friedewald formula, S-LDL-C= LDL-C calculated using Sampson formula, A-LDL-C = LDL-C calculated using Anandaraja formula, MH-LDL-C = LDL-C calculated using Martin-Hopkins formula

	MD (95%CI)±1.96SD
d-LDL-C and F-LDL-C	0.179 (0.049-0.310)±2.15
d-LDL-C and S-LDL-C	-0.032 (-0.137-0.072)±1.72
d-LDL-C and A-LDL-C	0.458 (0.340-0.576)±1.94
d-LDL-C and MH-LDL-C	-0.678 (-0.802-(-0.533)±2.04

**Figure 3:**
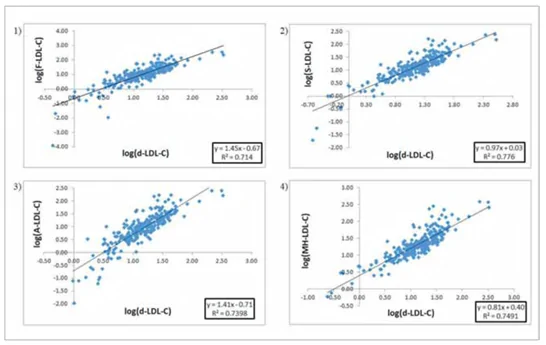
Linear regression analysis between d-LDL-C and 1) F-LDL-C, 2) S-LDL-C, 3) A-LDL-C, 4) MH-LDL-C.

Figure 3 presents the linear regression analyses of log-transformed data showed that the intercept was determined as -0.67 (95%CI=-0.81 to -0.54), 0.03 (95%CI=-0.05 to 0.10), -0.71 (95%CI=-0.83 to -0.59) and 0.40 (95%CI=0.36 to 0.47) and slopes were calculated as 1.45 (95%CI = 1.34-1.56), 0.97 (95%CI = 0.91—1.04), 1.41 (95%CI = 1.31-1.51) and 0.81 (95%CI=0.75-0.86) for F-LDL-C, S-LDL-C, A-LDL-C and MH-LDL-C, respectively.

Table 3 shows median and interquartile range (Q1-Q3) for LDL-C values measured with the direct method as well as those calculated using formulas. The table includes the P-value obtained from comparing d-LDL-C with the calculated LDL-C. All formulas, except for the S-LDL-C, showed a statistically significant difference compared to d-LDL-C. Post hoc analysis revealed statistically significant differences among all LDL-C values calculated using the formulas (P<0.001).

**Table 3:** Comparison of d-LDL-C with calculated LDL-C values. d-LDL-C = low-density lipoprotein-cholesterol determined by direct method, F-LDL-C = LDL-C calculated using Friedewald formula, S-LDL-C = LDL-C calculated using Sampson formula, A-LDL-C = LDL-C calculated using Anandaraja formula, MH-LDL-C = LDL-C calculated using Martin-Hopkins formula, *-Friedman test, Conover's post hoc comparisons method

	n = 272 <br>Me (IQR)	P-value*
d-LDL-C (mmol/L)	3.26 (2.52-4.14)	-
F-LDL-C (mmol/L)	2.89 (1.96-3.88)	<0.001
S-LDL-C (mmol/L)	3.17 (2.44-3.98)	0.240
A-LDL-C (mmol/L)	2.68 (1.82-3.64)	<0.001
MH-LDL-C (mmol/L)	3.69 (2.95-4.58)	<0.001

The calculated LDL-C values obtained using each formula were compared with the respective d-LDL-C values, according to categories representing target values based on cardiovascular risk assessment (Table 4). The overall misclassification rate were as follows: 41.9% (F-LDL-C), 32.7% (S-LDL-C), 50.0% (A-LDL-C) and 34.2% (MH-LDL-C).

**Table 4:** Agreement between calculated and d-LDL-C values based on guideline-defined categories. d-LDL-C=direct low-density lipoprotein cholesterol, F-LDL-C=LDL-C calculated using Friedewald formula, S-LDL-C=LDL-C calculated using Sampson formula, A-LDL-C=LDL-C calculated using Anandaraja formula, MH-LDL-C = LDL-C calculated using Martin-Hopkins formula

		d-LDL-C (mmol/L) <br>n = 272				
		< 1.4 <br>n = 13	1.4-1.8 <br>n = 14	1.8-2.6 <br>n=48	2.6-3.0 <br>n=43	>3.0 <br>n = 154
		% (N)				
< 1.4 <br>mmol/L	F-LDL-C	4.78 (13)	2.94 (8)	3.68 (10)	0.39 (1)	0.39 (1)
	S-LDL-C	2.94 (8)	1.47 (4)	0 (0)	0 (0)	0 (0)
	A-LDL-C	4.41 (12)	3.68 (10)	4.41 (12)	0.39 (1)	0 (0)
	MH-LDL-C	1.10 (3)	0 (0)	0 (0)	0 (0)	0 (0)
1.4-1.8 <br>mmol/L	F-LDL-C	0 (0)	1.47 (4)	4.41 (12)	1.84 (5)	0.39 (1)
	S-LDL-C	1.84 (5)	0.39 (1)	1.84 (5)	0 (0)	0 (0)
	A-LDL-C	0.39 (1)	1.10 (3)	5.52 (15)	3.31 (9)	1.10 (3)
	MH-LDL-C	1.84 (5)	1.10 (3)	0 (0)	0 (0)	0 (0)
1.8-2.6 <br>mmol/L	F-LDL-C	0 (0)	0.39 (1)	6.99 (19)	7.72 (21)	6.25 (17)
	S-LDL-C	0 (0)	2.94 (8)	11.03 (30)	5.51 (15)	2.21 (6)
	A-LDL-C	0 (0)	0 (0)	6.25 (17)	7.35 (20)	9.56 (26)
	MH-LDL-C	1.84 (5)	2.57 (7)	5.88 (16)	0.39 (1)	0.39 (1)
2.6-3.0 <br>mmol/L	F-LDL-C	0 (0)	0 (0)	1.84 (5)	2.94 (8)	7.72 (21)
	S-LDL-C	0 (0)	0 (0)	2.94 (8)	4.78 (13)	6.25 (17)
	A-LDL-C	0 (0)	0.39 (1)	0.74 (2)	2.21 (6)	9.93 (27)
	MH-LDL-C	0 (0)	1.10 (3)	5.15 (14)	3.30 (9)	1.84 (5)
>3.0 <br>mmol/L	F-LDL-C	0 (0)	0.39 (1)	0.74 (2)	2.94 (8)	41.91 (114)
	S-LDL-C	0 (0)	0.39 (1)	1.84 (5)	5.52 (15)	48.16 (131)
	A-LDL-C	0 (0)	0 (0)	0.74 (2)	2.57 (7)	36.03 (98)
	MH-LDL-C	0 (0)	0.39 (1)	6.62 (18)	12.13 (33)	54.41 (148)

## Discussion

In this study, we evaluated the performance of four commonly used LDL-C estimation formulas in patients with hypertriglyceridemia and compared their results with d-LDL-C values. Our findings demonstrated a strong positive correlation between all calculated LDL-C values and d-LDL-C, with the Sampson formula showing the highest level of agreement and the lowest MD.

In our study, LDL-C values calculated using four different formulas showed a statistically significant linear correlation with d-LDL-C levels (P<0.001) in patients with hypertriglyceridemia (>4.5 mmol/L), consistent with findings from other studies comparing d-LDL-C and LDL-C calculated using various formulas [3][6][11][12][13][14]. Among the tested equations, A-LDL-C exhibited the highest correlation coefficient (r = 0.867), suggesting relatively strong predictive performance in this population. However, despite this high correlation, A-LDL-C and F-LDL-C formulas tended to overestimate LDL-C levels compared with d-LDL-C, indicating the presence of proportional bias. In contrast, MH-LDL-C showed a consistent underestimation, while S-LDL-C demonstrated the best agreement with d-LDL-C, characterized by a slope not significantly different from 1 (0.97, p=0.53) and minimal bias (0.03).

Based on our findings, the formula proposed by Sampson and colleagues performed best among the tested equations, consistent with previous reports indicating superior accuracy in patients with elevated triglyceride levels. It showed superior intercept and slope values, the lowest MD, and was the only one that did not show statistically significant differences compared with the d-LDL-C. The superior performance of the S-LDL-C can be explained by its more physiologically grounded approach to lipid metabolism. S-LDL-C was derived from a large dataset using direct b-quantification measurements and incorporates a more accurate nonlinear relationship between TG, VLDL, and LDL-C [8]. This allows better estimation of LDL-C, especially in individuals with elevated TG levels or altered lipid metabolism [8]. The formula better reflects the variable composition of VLDL particles and their changing CHOL-to-TG ratio across different metabolic states [15]. Therefore, its improved correlation with d-LDL-C likely reflects a more realistic modeling of lipoprotein metabolism. In our study, although it gives slightly higher results than the direct method (MD=-0.032), the 95% CI includes zero, indicating no statistically significant difference between the two methods. The regression slope of 0.973 suggests a mild proportional bias, with lower calculated values observed at higher LDL-C concentrations. This pattern indicates that the S-LDL-C slightly overestimates LDL-C at lower concentrations and underestimates it at higher concentrations, consistent with minor proportional deviation but minimal overall bias. According to the authors, the misclassification rate in patients with hypertriglyceridemia was 35% lower than that with the Friedwald equation [8]. In our study, the Sampson formula demonstrated a misclassification rate of 32.7%.

In contrast, F-LDL-C has several limitations. It requires separate analyses of CHOL, TG, and HDL-C, so methodological errors can accumulate. Additionally, the formula assumes that the ratio of CHOL to TG in VLDL particles is constant, making it unsuitable for use when TG concentrations in the serum exceed 4.5 mmol/L [2]. It also tends to overestimate or underestimate LDL-C levels in individuals with conditions such as diabetes mellitus, alcoholic liver disease, or chronic renal failure [3]. In our study, we applied the Friedewald formula to patients with elevated TG levels to compare its effectiveness with other proposed formulas. The observed overestimation by the A-LDL-C and underestimation by the MH-LDL-C further emphasize that no single equation performs uniformly across all lipid profiles, and formula-specific biases may depend on population characteristics and triglyceride distribution.

In various studies conducted in different population groups, different results were obtained compared to our findings. One of those studies conducted in the Italian population found that the highest degree of correlation was shown by S-LDL-C in all subjects, whereas among subjects with triglyceride levels above 4.5 mmol/L, a higher correlation coefficient was observed for the MH-LDL-C [6]. Study of Piani and colleagues showed that S-LDL-C resulted as most accurate equation for LDL-C estimation with lowest underestimation rates, similar to our results where the formula of Sampson et al. showed the lowest MD [6]. Considering all TG levels, the overall concoradance in this study was 85.7% for S-LDL-C, compared to MH-LDL-C 83.8%, 76.8% for F-LDL-C, and 70.5% for A-LDL-C [6]. This study evaluated 12 formulas, unlike ours, where we evaluated four formulas. By comparing d-LDL-C and LDL-C obtained by calculation, in our study only formula that did not show a significant difference in the examined population was the S-LDL-C (P=0.240).

Another study evaluating nine different LDL-C estimation formulas at a tertiary care center in Hyderabad, India, found that the A-LDL-C exhibited the highest degree of correlation among subjects with TG values over 4.5 mmol/L, as well as ours study. Formulas according to Friedwald, Anandaraja, and Martin-Hopkins were found to be highly inaccurate [3]. Notably, Sampson's formula was not assessed in the study by Sirivelu and colleagues [3].

A study by Wadhwa and colleagues, also conducted in India, showed that in a patient population with TG levels above 4.5 mmol/L F-LDL-C and A-LDL-C, the same as in our study showed significant differences compared to d-LDL-C [13].

The accuracy of lipoprotein measurements is essential for the prevention of ACVD and clinical decisions depend on the availability of accurate and reproducible laboratory measurements [4]. Our results showed that the Sampson aquation (32.7%) and the Martin-Hopkins equation (34.2%) had the lowest misclassification rates compared to the Anandaraja (50.0%) and Friedewald (41.9%) formulas. While the Sampson equation demonstrated the lowest misclassification rate among the evaluated formulas, it still misclassified nearly one-third of patients, a limitation that could have important implications for clinical decision-making and treatment planning. Measurement accuracy is important to avoid adverse outcomes for patients [3]. Underestimating or overestimating LDL-C values can lead to a delays in therapy or unnecessary exposure of patients to medications. While LDL-C can be directly measured using enzymatic techniques, in practice it is most frequently calculated using the Friedewald formula [3].

This study has certain limitations. The main limitations include the relatively small number of examined patients and the limited number of LDL-C estimation formulas evaluated. In addition, LDL-C measurement by beta-quantification, which represents the gold standard method, was not performed. As this was a single-center, retrospective study, the generalizability of the results may be limited. Despite these constraints, our findings provide useful insight into the performance of the tested LDL-C formulas in our population and may contribute to the optimization of LDL-C estimation in routine clinical practice.

## Conclusion

In the studied population of patients with hypertriglyceridemia the formula by Sampson et al. [15], demonstrated the best overall performance, showing the closest agreement with the direct method and was the only one that did not show a statistically significant difference compared to the direct method. However, it still misclassified approximately one-third of patients according to the recommended target values based on cardiovascular risk assessment, which may have implications for clinical decision-making and therapy adjustment. These findings suggest that the Sampson formula may be the most applicable alternative to direct LDL-C measurement in resource-limited settings, although direct methods remain preferable for patients with elevated triglyceride levels. Further studies on larger and more diverse populations are recommended to validate these results and refine LDL-C estimation formulas for clinical use.

## Dodatak

### Conflict of interest statement

All the authors declare that they have no conflict of interest in this work.

### List of abbreviations:

CHOL, cholesterol; <br>TG, triglycerides; <br>LDL-C, low-density lipoproteins cholesterol; <br>d-LDL-C, LDL-cholesterol determined by direct method; <br>ACVD, atherosclerotic cardiovascular disease; <br>F-LDL-C, LDL-C calculated using Friedewald equation; <br>S-LDL-C, LDL-C calculated using Sampson-National Institutes of Health Equation 2; <br>A-LDL-C, LDL-C calculated using Anandaraja formula; <br>MH-LDL-C, LDL-C calculated using Martin-Hopkins formula; <br>HDL-C, high-density lipoproteins -cholesterol; <br>VLDL, very low-density lipoproteins
